# Recent Advances in Natural Extract-Based Multifunctional Gels for Food Packaging: A Review

**DOI:** 10.3390/gels12080679

**Published:** 2026-08-01

**Authors:** Jiayi Xue, Rui Zhang, Wanxiang Xu, Haoqiang Wang, Congyu Lin, Yuan Fu, Yingzhu Liu, Longwei Jiang

**Affiliations:** 1 Key Laboratory of Jianghuai Agricultural Product Fine Processing and Resource Utilization of Ministry of Agriculture and Rural Affairs, Anhui Engineering Research Center for High Value Utilization of Characteristic Agricultural Products, School of Food and Nutrition, Anhui Agricultural University, Hefei 230036, China; xuejiayi0205@163.com (J.X.); 18052700620@163.com (W.X.); w1713545068@163.com (H.W.); 2 School of Forestry and Landscape Architecture, Anhui Agricultural University, Hefei 230036, China; 13293953968@163.com; 3 School of Food Science and Technology, Jiangnan University, Wuxi 214122, China; lincongyu@jiangnan.edu.cn; 4 School of Biological Science and Food Engineering, Chuzhou University, No. 1, West Huifeng Road, Chuzhou 239099, China; 5 Laboratory of Organic Chemistry, Wageningen University, 6708 WE Wageningen, The Netherlands

**Keywords:** natural extracts, multifunctional gels, matrix composition, food packaging, applied research

## Abstract

Food packaging plays an essential role in maintaining food quality, safety, and shelf life during storage, transportation, and retail distribution. However, conventional petroleum-derived plastics raise increasing environmental concerns, while many biopolymer-based packaging materials remain limited by insufficient mechanical strength, poor water resistance, limited barrier properties, and unstable active functions. Natural extract-based multifunctional gels provide a promising strategy for sustainable food packaging due to their inherent biocompatibility, structural versatility, and synergistic interplay between active extracts and gel networks, which can immobilize bioactive compounds, regulate mass transfer, and integrate preservation and monitoring functions within one material platform. This review summarizes recent advances in natural extract-based multifunctional gels for food packaging, with emphasis on polysaccharide-based, protein-based, lipid-based, and other naturally derived gel systems. Representative material formats include hydrogels, aerogels, emulsion gels, oleogels, and gel-derived films or coatings, while typical components include chitosan, alginate, pectin, starch, proteins, natural waxes, lignin, anthocyanins, curcumin, polyphenols, and essential oils. Their gelation mechanisms, structural features, preparation strategies, and functional applications are discussed, including antioxidant activity, antimicrobial preservation, barrier enhancement, controlled release, freshness monitoring, and biodegradability. Furthermore, the potential interactions between natural extracts and gel matrices are also discussed, including hydrogen bonding, electrostatic interactions, ionic crosslinking, dynamic covalent bonding, hydrophobic association, and lipid crystallization. The main merits of these systems include renewability, biodegradability, tunable network interactions, active-agent protection, and integration of preservation and monitoring functions, whereas persistent challenges include moisture sensitivity, mechanical instability, variable extract composition, migration safety, and scale-up cost. This review provides guidance for designing natural extract-based gel packaging materials with improved functionality, sustainability, and practical applicability.

## 1. Introduction

Food products are highly susceptible to microbial contamination, oxidative reactions, moisture migration, enzymatic browning, and mechanical damage during storage, transportation, and retail distribution, resulting in nutritional degradation, sensory quality deterioration, and reduced shelf life. Conventional petroleum-based plastic packaging materials offer satisfactory mechanical strength, barrier performance, and processing convenience; however, their reliance on non-renewable resources, poor degradability, and contribution to microplastic pollution have raised increasing environmental concerns. In response to these challenges, gel-based packaging systems constructed from natural polymers and natural extracts have become an important structural platform for sustainable food packaging. Unlike the simple addition of active compounds, gel networks can immobilize bioactive substances, regulate water/oil phases and mass transfer, and provide mechanical or barrier support during food storage [1,2,3]. Agricultural and food-processing by-products can supply polysaccharides, proteins, lipids, and bioactive extracts, but composition, contaminants, migration, and toxicological safety must be verified before food-contact use.

**Figure 1:**
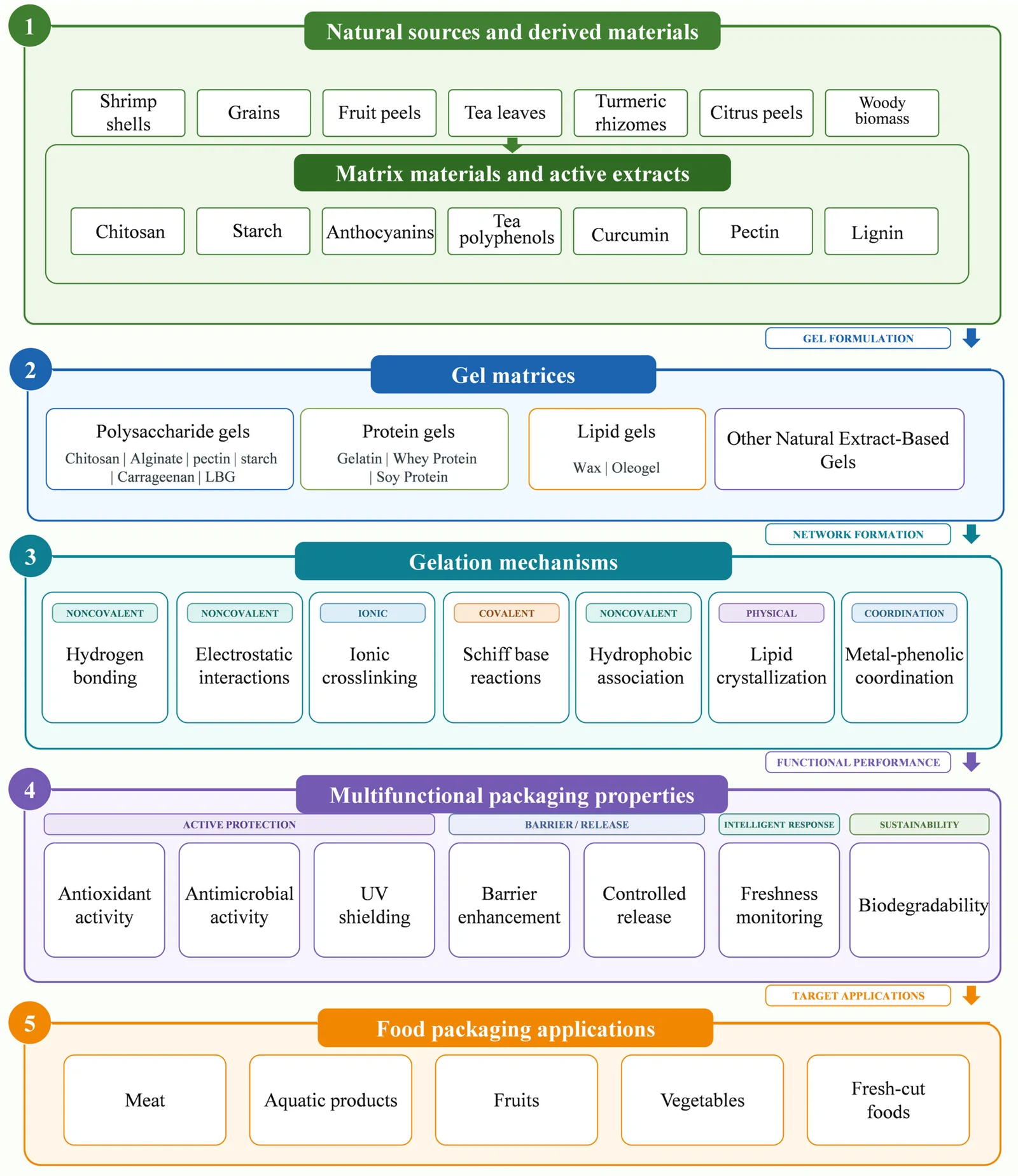
Overview of natural extract-based multifunctional gels for food packaging.

As shown in Figure 1, natural resources such as shrimp shells, seaweed, fruit peels, cereals, tea leaves and turmeric rhizomes can supply active compounds, whereas wood-derived lignocellulosic fractions can provide matrix or functional components for multifunctional gel packaging. Chitosan, alginate, pectin, starch, carrageenan, gelatin, waxes, lignin, and related biopolymers mainly construct the gel network, providing film-forming ability, mechanical support, barrier regulation, and sites for active-agent immobilization. Anthocyanins, curcumin, tea polyphenols, flavonoids, tannic acid, and fruit and vegetable by-product extracts are commonly incorporated into these networks to provide antioxidant, antimicrobial, ultraviolet-shielding, pH-responsive, or volatile amine-responsive functions [4,5,6]. Therefore, the key issue in this review is not only what natural extracts can do, but how gel matrices organize, stabilize, and control these extracts in food packaging environments.

Depending on the continuous phase and processing route, these materials may be prepared as hydrogels, aerogels, oleogels, emulsion gels, or gel-derived films and coatings. Hydrogels are water-swollen networks suited to absorption and diffusion-controlled release; aerogels retain a dry, highly porous structure that accelerates gas and volatile-metabolite transport; oleogels immobilize liquid oil and improve the retention of lipophilic active compounds; emulsion gels integrate aqueous and oil phases; and drying or coating converts gel precursors into practical films, labels, pads, and edible surface layers. Thus, the term “gel” in this review covers both hydrated networks and gel-derived packaging formats.

From the perspective of material structure, the construction of gel networks is critical for achieving functionalization in natural extract-based packaging materials. Natural polymer chains or low-molecular-weight gelators can form three-dimensional networks through intermolecular interactions and assembly processes, including hydrogen bonding, electrostatic interactions, metal coordination, dynamic covalent bonding, hydrophobic association, lipid crystallization, and chain entanglement [2,3,7]. This network not only provides structural support for packaging materials, but also immobilizes active components and regulates their release, migration, and responsive behavior during food storage.

Previous reviews have discussed biodegradable packaging derived from food-processing by-products [1], the incorporation of natural extracts into gel systems [2], general hydrogel applications in foods [3,7], and active or intelligent packaging based on natural colorants and polyphenols [4,5,6]. The present review places greater emphasis on the role of the gel matrix in organizing and regulating natural active components. Recent studies are discussed according to polysaccharide-, protein-, lipid-, and other naturally derived matrices, followed by an analysis of how gelation mechanisms and network interactions influence swelling, active-component retention and release, barrier performance, and responsive behavior. These structure–function relationships are further linked to the preservation and monitoring requirements of meat, aquatic products, fruits, vegetables, and other food systems. Practical issues, including biodegradability, food-contact safety, processing feasibility, and industrial scale-up, are also considered. Therefore, this review provides a systematic understanding of how matrix composition and network structure determine the performance of natural extract-based gel packaging.

**Figure 2:**
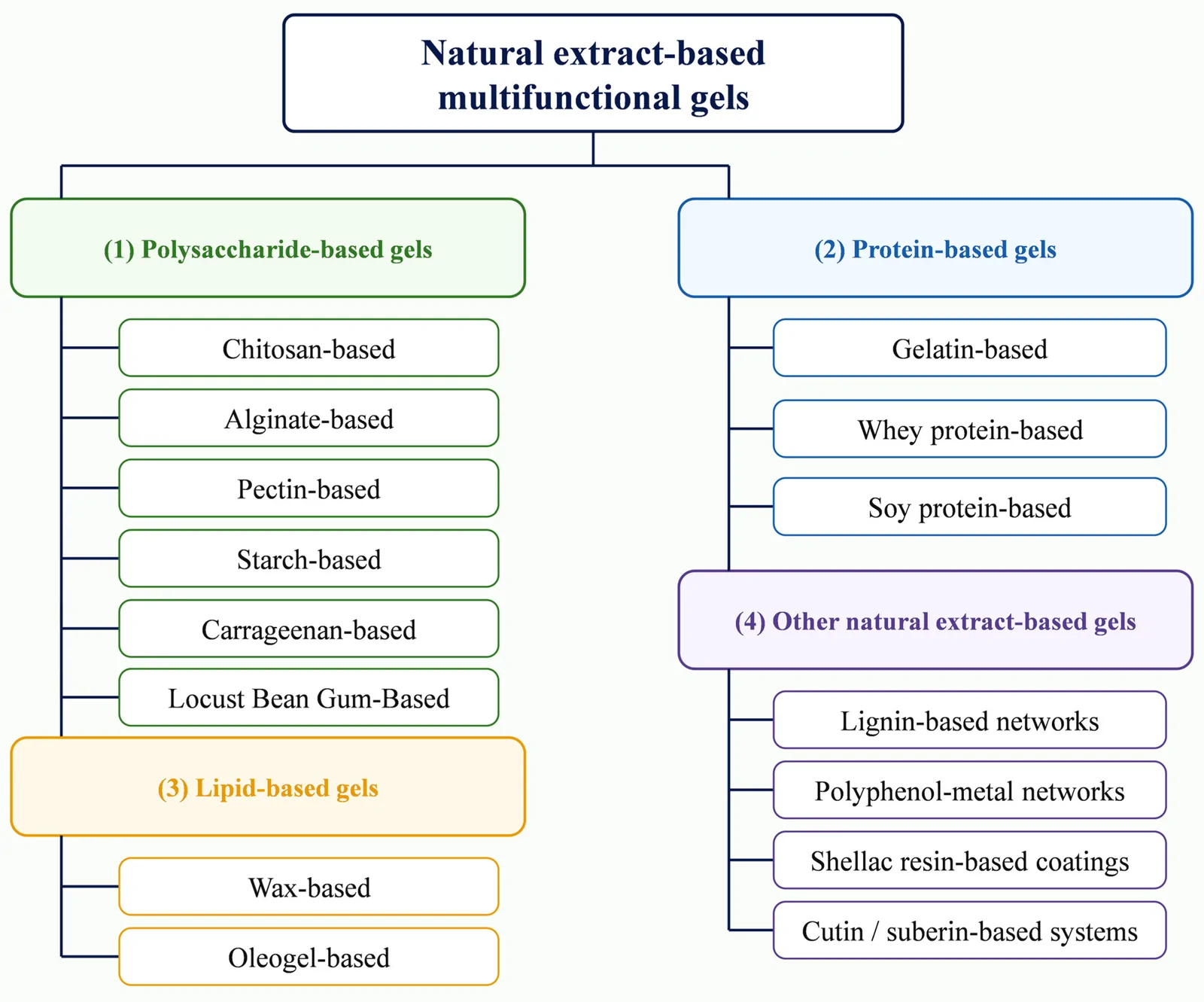
Classification of natural extract-based multifunctional gel matrices.

This review focuses on recent advances in natural extract-based multifunctional gels for food packaging. According to the type of matrix, the relevant systems are classified into four categories: polysaccharide-based gels, protein-based gels, lipid-based gels, and other naturally derived gels, as illustrated in Figure 2. Subsequently, the gelation mechanisms, preparation strategies, food preservation applications, and freshness monitoring functions of each gel system are discussed, followed by an analysis of their industrialization challenges and future development directions.

## 2. Polysaccharide-Based Gels

Polysaccharide-based gels are the most widely investigated natural gel matrices for food packaging because polysaccharide chains contain abundant hydroxyl, carboxyl, amino, or sulfate groups that can participate in ionic crosslinking, hydrogen bonding, Schiff base reactions, and chain entanglement. According to matrix structure and gelation behavior, five common polysaccharide-based gel systems are discussed in this section, including chitosan-, alginate-, pectin-, starch-, and carrageenan-based gels.

### 2.1. Chitosan-Based Gels

Chitosan is one of the most widely used polysaccharide matrices in food packaging gels. Its molecular chains are rich in amino and hydroxyl groups, endowing chitosan with favorable film-forming ability, biocompatibility, biodegradability, and inherent antimicrobial activity to some extent [8,9,10,11]. Under acidic conditions, the amino groups of chitosan are readily protonated to –NH_3_^+^, giving chitosan typical cationic polysaccharide characteristics. As a result, it can interact with anionic polysaccharides, natural polyphenols, anthocyanins, and other functional components to form composite gel networks through electrostatic interactions, hydrogen bonding, and hydrophobic interactions. In addition, the amino groups of chitosan can undergo Schiff base reactions with aldehyde-containing compounds to form dynamic C=N covalent bonds, thereby enhancing network stability and the immobilization capacity for active agents. Recent studies on chitosan-based packaging gels have mainly focused on two application directions: active preservation and intelligent indication. Flavonoids, tea polyphenols, and curcumin can endow chitosan films with antioxidant and antimicrobial activities for the preservation of foods such as beef. For example, the preservation performance of chitosan–flavonoid films for beef was evaluated at 4 °C for two weeks, and the composite-film-packaged group exhibited a lower total viable count than the commercial fresh-keeping film group. In another study, chitosan/polyvinyl alcohol films containing curcumin inclusion complexes showed 2,2′-azinobis-(3-ethylbenzothiazoline-6-sulfonic acid) (ABTS) and 2,2-diphenyl-1-picrylhydrazyl (DPPH) radical-scavenging activities of 98% and 87%, respectively, and reduced the viable counts of *Staphylococcus aureus* (*S. aureus*) and *Escherichia coli* (*E. coli*) [8,9]. Anthocyanins and curcumin can also serve as pH- and volatile amine-responsive components for freshness monitoring of meat and aquatic products [10,11]. Meanwhile, chitin nanofibers can enhance the mechanical properties and structural stability of chitosan films [12,13]. Chitin nanofiber-reinforced chitosan/curcumin aerogels have also been developed for real-time freshness monitoring of beef. In particular, the aerogel containing 5 g/100 g chitin nanofibers exhibited a porosity of over 98%, a density below 0.02 g cm^−3^, and a 118% increase in compressive strength compared with the aerogel without chitin nanofibers. Its highly porous structure facilitates sufficient contact between volatile amines and the indicator molecules [14].

Chitosan is also widely used as a direct edible coating, where its function differs from that of a detached indicator film. Coating performance is governed by the degree of deacetylation, molecular weight, concentration, acid solvent, and coating thickness. Chitosan coatings helped preserve organic acids and bioactive compounds in mandarins [15]. A chitosan hydrochloride/gum arabic coating carrying passion fruit seed oil nanoparticles suppressed fungal infection and maintained strawberry quality at both 25 and 4 °C [16]. Water-soluble chi-tosan/curdlan coatings also improved cherry tomato preservation and inhibited postharvest pathogenic fungi [17]. However, excessive coating density may restrict respiration or alter sensory properties; therefore, composition and thickness should be optimized for each food.

### 2.2. Alginate-Based Gels

Alginate is mainly derived from brown algae and consists of beta-D-mannuronic acid and alpha-L-guluronic acid units, making it a typical anionic polysaccharide matrix [18]. Its most representative gelation mechanism is the “egg-box” model, in which consecutive guluronic acid blocks in the molecular chains coordinate with divalent cations such as Ca^2+^ and Zn^2+^, thereby improving gel integrity and wet-state stability. Alginate can also form hydrogen-bonded composite networks with cellulose nanocrystals, carboxymethyl cellulose, guar gum, polyvinyl alcohol, pectin, and other polymers, which can be used to immobilize natural active substances such as anthocyanins, curcumin, and vegetable oil emulsions. Recent studies have shown that sodium alginate/anthocyanin/cellulose nanocrystal films modified with beeswax or a hydrophobic nanosilica layer can significantly improve the water resistance and food-monitoring stability of indicator films. For example, a 5% beeswax coating increased the water contact angle to 111.5°, and the film still maintained a contact angle of 108.3° after storage for 10 d at 100% relative humidity [19,20,21]. Curcumin-sodium alginate-polyvinyl alcohol films can be used for shrimp freshness monitoring. For instance, the composite film containing 30 mg curcumin changed from light yellow to purple on the fourth day of shrimp storage, with the total color difference increasing from 34.52 to 51.89 [22]. In addition, incorporating a Pickering emulsion into sodium alginate/guar gum films can enable sustained release of oil-phase active substances and has been applied for mushroom preservation [23]. Furthermore, carboxymethyl cellulose/sodium alginate aerogel colorimetric labels can accelerate volatile amine response through their porous structure [24].

### 2.3. Pectin-Based Gels

Pectin is widely distributed in plant cell walls and fruit and vegetable processing by-products. It is mainly composed of galacturonic acid units and exhibits good film-forming ability, gelation capacity, edibility, and biodegradability [25,26]. Low-methoxyl pectin contains abundant free carboxyl groups and can form egg-box-like ionic crosslinked networks with metal ions such as Ca^2+^, Zn^2+^, and Fe^3+^, whereas high-methoxyl pectin generally gels through hydrogen bonding and hydrophobic aggregation under acidic and high-sugar conditions. In recent years, pectin has often been combined with alginate, starch, carboxymethyl cellulose, whey protein, or gelatin to improve film mechanical properties, water stability, and active-agent immobilization. Pectin/alginate/cellulose nanocrystal/anthocyanin intelligent films can be used for food freshness monitoring [25]. Metal cation crosslinking can regulate the structural compactness and shrimp freshness-monitoring sensitivity of pectin/carboxymethyl cellulose sodium/anthocyanin films, with the correlation coefficients between total color difference and shrimp pH or total volatile basic nitrogen both exceeding 0.98 in Zn^2+^-crosslinked films [26]. A dragon fruit peel pectin/cassava starch system combined with cyanidin and alizarin can indicate pork freshness and demonstrates the value-added utilization of fruit and vegetable by-products [27]. In addition, polysaccharide-polysaccharide or protein-polysaccharide networks constructed from pectin with starch, whey protein, or gelatin can be used for freshness detection of chilled meat, grapes, shrimp, and other meat products. For example, pectin/whey protein films containing purple sweet potato extract showed the lowest water solubility and water vapor permeability at a pigment addition level of 500 mg/100 mL [28,29,30].

### 2.4. Starch-Based Gels

Starch is composed of amylose and amylopectin. It is abundant, low-cost, renewable, and biodegradable, making it one of the most promising natural polysaccharides for food packaging [31]. Starch-based gels mainly form networks through gelatinization, chain rearrangement, and retrogradation. During heating, starch granules absorb water and swell, crystalline regions are disrupted, and amylose is released; during cooling and drying, molecular chains rearrange through hydrogen bonding to form film structures dominated by physical crosslinking and chain entanglement [31,32]. Because starch films are often limited by high brittleness and strong water sensitivity, they are often blended with polyvinyl alcohol, carboxymethyl cellulose, alginate, pectin, nanocellulose, and other materials. *Dioscorea zingiberensis* starch/anthocyanin films and carboxymethyl cellulose/sodium alginate/starch/anthocyanin/tea polyphenol composite films can be used for meat freshness monitoring [32,33]. Incorporating *Clitoria ternatea* flower or purple sweet potato peel extracts into starch/polyvinyl alcohol films can simultaneously enable antioxidant release, pH response, and composability. For example, films containing *Clitoria ternatea* extract showed an average weight loss of approximately 70% during composting, while jackfruit seed starch/polyvinyl alcohol/purple sweet potato peel anthocyanin films exhibited color changes from red to yellow over the pH range of 1–13 [34,35]. Ganyong starch (*Canna edulis* Kerr.) films modified with corn husk nanocellulose and curcumin further demonstrate the utilization potential of agricultural by-products in intelligent packaging materials [36].

### 2.5. Carrageenan-Based Gels

Carrageenan is a sulfated galactan extracted from red algae, and its common types include kappa (κ)-carrageenan, iota (ι)-carrageenan, and lambda (λ)-carrageenan. Its gel formation mainly depends on thermally induced conformational transition and cation-induced double-helix aggregation. Upon heating, carrageenan chains exist as random coils; upon cooling, they form ordered double helices, which further aggregate into three-dimensional networks in the presence of K^+^ or Ca^2+^. Carrageenan has good transparency, edibility, and compatibility with natural pigments, making it suitable for constructing intelligent indicator films [37]. In recent years, anthocyanins and TiO_2_-doped carbon dots derived from sweet potato peels, kohlrabi peel-derived anthocyanins and ZnO-doped carbon dots, curcumin, epigallocatechin gallate (EGCG), carbon dot-silica nanohybrids, and gromwell root extract rich in shikonin have been incorporated into carrageenan or carrageenan/gelatin composite films for preservation and freshness monitoring of shrimp, meat, and aquatic products. For example, a carrageenan film containing kohlrabi peel-derived anthocyanins and ZnO-doped carbon dots showed UV-A/UV-B blocking rates of 85.2%/99.4% and reduced *E. coli* by 8.1 log CFU/mL. A κ-carrageenan-based film reinforced with curcumin-anthocyanin-EGCG nanoparticles reduced water vapor permeability, while the radical scavenging activity increased from 1.75% to 77.65% [37,38,39,40,41]. Overall, these systems are commonly prepared by casting, where natural pigments provide pH-responsive ability, whereas carbon dots or nanofillers further enhance optical, antimicrobial, antioxidant, and UV-shielding properties.

### 2.6. Locust Bean Gum-Based Gels

Locust bean gum (LBG), or carob gum, is a neutral galactomannan extracted from the endosperm of Ceratonia siliqua seeds. It consists of a β-(1 → 4)-linked mannose backbone with α-(1 → 6)-linked galactose side groups. Its abundant hydroxyl groups support hydration, hydrogen bonding, and film formation, but LBG alone usually produces highly hydrophilic films with limited mechanical and barrier properties [42]. More importantly, the less-substituted mannan regions associate synergistically with xanthan gum or carrageenan to form denser physical gel networks; recent atomic force microscopy analysis clarified the assembly of LBG/xanthan gum at the molecular scale [43]. LBG can also form composite matrices with chitosan, alginate, and carboxycellulose nanocrystals. Chitosan/LBG films containing *Koelreuteria paniculata* Laxm. bract extract and Ti-doped carbon dots combined antioxidant and antimicrobial preservation with pH-responsive shrimp freshness monitoring [44]. In another study, carboxycellulose nanocrystals increased the tensile strength and elongation at break of LBG films by 43.69% and 49.13%, respectively, while the resulting coating reduced strawberry weight loss by 19.42% [45]. Thus, LBG is most effective as a co-matrix or coating former whose performance is adjusted through synergistic polysaccharide association or nanofiber reinforcement.

Other polysaccharides, including konjac glucomannan, xanthan gum, gellan gum, pullulan, curdlan, gum arabic, guar gum, and cellulose derivatives, further broaden the available gel and coating matrices. Konjac glucomannan/xanthan gum formed a hydrogen-bonded self-healing hydrogel coating that delayed banana water loss and browning and extended shelf life by more than 6 d [46]. Gellan gum can form ion-set coatings and carry oregano essential oil for mandarin preservation [47], whereas pullulan films carrying biocontrol yeast reduced multiple citrus molds without compromising major quality parameters [48]. These matrices should be selected according to the required gelation route, adhesion, gas permeability, active-agent release, and food surface rather than treated as interchangeable film formers.

Overall, polysaccharide-based gels are attractive because of their low cost, biodegradability, transparency, and compatibility with natural pigments and polyphenols. However, their strong hydrophilicity remains a major limitation: many systems show poor wet-state strength, high water vapor permeability, swelling, brittleness, or active-component leaching under high-humidity food conditions. In addition, the color response of polysaccharide indicator gels can be affected by matrix pH, ionic strength, pigment migration, and batch variability of natural extracts.

## 3. Protein-Based Gels

Proteins constitute another vital raw material for fabricating food packaging gels. Compared with polysaccharides, proteins contain more diverse amino acid residues and can form networks through triple-helix reconstruction, heat-induced unfolding, disulfide exchange, hydrophobic association, hydrogen bonding, and electrostatic interactions. These structures are especially useful for binding natural pigments, polyphenols, essential oils, and nanoparticles while improving oxygen barrier properties.

### 3.1. Gelatin-Based Gels

Gelatin is obtained from the partial hydrolysis of collagen and can be derived from sources such as pig skin, bovine bone, fish skin, and fish scales. It exhibits good film-forming ability, transparency, and thermoreversible gelation [49]. Its gel formation mainly relies on the partial reformation of collagen-like triple-helix structures during cooling, which shortens intermolecular distances and forms physical networks through hydrogen bonding and chain entanglement. Natural pigments and polyphenols can form hydrogen bonds or electrostatic interactions with amino, carboxyl, and hydroxyl groups in gelatin, thereby improving the immobilization of active substances. Recent studies have shown that anthocyanin-enriched wheat gluten/gelatin electro-spun films can enable real-time shrimp freshness detection [49]. These films responded to acetic acid and ammonia within 40 s. The correlation coefficients between total color difference and shrimp total volatile basic nitrogen and total viable count were 0.988 and 0.962, respectively. Gelatin/pectin composite films incorporated with *Hibiscus sabdariffa* L. (roselle) anthocyanins and orange peel-derived carbon dots can be used for meat freshness detection [30]. Zein-quercetin nanoparticle-reinforced gelatin films decreased water vapor and oxygen permeability by 78.4% and 76.9%, respectively. These films extended the shelf life of strawberries to approximately 8 d [50].

### 3.2. Whey Protein-Based Gels

Whey protein is mainly derived from dairy processing by-products and has good film-forming ability, nutritional value, and oxygen barrier properties. Its gelation usually requires heat-induced unfolding, which exposes sulfhydryl and hydrophobic groups in components such as β-lactoglobulin, followed by network formation through hydrophobic interactions, disulfide bond exchange, hydrogen bonding, and electrostatic interactions [51]. Pectin/whey protein composite pH-sensitive films loaded with purple sweet potato extract can be used for both grape preservation and shrimp freshness monitoring [29]. Whey protein isolate films containing lemon and bergamot essential oils can provide active preservation functionality and inhibit *S. aureus* and *E. coli* [51]. Whey-protein films are attractive for oxygen-sensitive foods because heat-induced networks can retain active compounds, but moisture sensitivity and allergen labeling require consideration [52].

### 3.3. Soy Protein-Based Gels

Soy protein is one of the widely used plant-derived proteins and is relatively low in cost. It mainly consists of β-conglycinin (7S) and glycinin (11S) globulins. Under heating, alkaline treatment, or crosslinking conditions, soy protein can undergo conformational unfolding, exposing hydrophobic regions and reactive groups, and then form gel-like films through hydrophobic interactions, hydrogen bonding, disulfide bonds, or Schiff base reactions [53,54]. *Zanthoxylum bungeanum* by-products and Tara pod extract can interact with soy protein through polyphenol-protein interactions, thereby improving antioxidant, barrier, and preservation properties. For example, soy protein isolate films containing 5% *Zanthoxylum bungeanum* leaf extract showed a tensile strength and elongation at break of 12.95 MPa and 85.33%, respectively [53,55]. Synergistic modification with dialdehyde polysaccharides and nanoparticles can strengthen the structure of soy protein films enriched with lingonberry extract [54]. Chitosan/soy protein isolate-based films incorporated with purple hull pistachio-derived carbon dots and anthocyanins can be used for fish preservation and freshness monitoring, with ABTS and DPPH radical scavenging activities of 82.3% and 90.6%, respectively, and the shelf life of fish extended to 12 d at 4 °C [56]. Soy lipophilic protein/hydroxypropyl methylcellulose pH-responsive films can also be used for salmon freshness monitoring [57].

Nevertheless, protein-based gels also have clear weaknesses. Gelatin, whey protein, and soy protein films are generally sensitive to moisture, and their mechanical and barrier properties may decline sharply under high relative humidity. Protein sources may also involve cost, allergenicity, odor, religious or dietary restrictions, and batch stability issues. Moreover, strong protein-polyphenol or protein-pigment binding can improve immobilization but may reduce release efficiency or weaken colorimetric sensitivity. These limitations indicate that protein-based gels require careful regulation of crosslinking density, plasticizer level, and active-agent binding strength.

## 4. Lipid-Based Gels

Lipid-based gels differ from polysaccharide and protein gels in that their main contribution is hydrophobic barrier formation and lipophilic active-agent retention. Wax crystallization, oleogelator self-assembly, and lipid network formation can reduce water vapor transfer and provide reservoirs for essential oils, carotenoids, polyphenols, and other hydrophobic natural extracts.

### 4.1. Wax-Based Gels

Wax-based gels or wax-based coatings usually use natural waxes such as beeswax, carnauba wax, candelilla wax, and rice bran wax as the main lipid components. These waxes are mainly composed of long-chain fatty acid esters, hydrocarbons, fatty alcohols, and free fatty acids, and they exhibit strong hydrophobicity and low water vapor permeability. Therefore, they are commonly used as fruit and vegetable surface coatings, hydrophobic layers for paper-based packaging, and barrier-enhancing layers for hydrophilic biofilms [58]. Their gelation or film formation generally involves melting, emulsification, cooling crystallization, and drying. Molten waxes are dispersed as wax droplets in the presence of polysaccharides, proteins, or emulsifiers, and then form dispersed or continuous hydrophobic phases through cooling crystallization and water evaporation. This structure fills pores in the film matrix, extends the diffusion path of water and gases, and reduces the loss of volatile active substances. Recent studies have shown that arrowroot starch/beeswax-based coatings loaded with clove essential oil can reduce weight loss in tomatoes, delay changes in color and texture, and inhibit microbial growth. In particular, an arrowroot starch coating loaded with clove essential oil and beeswax significantly delayed tomato quality deterioration during 48 d of storage at 26 ± 2 °C and 72 ± 2% relative humidity [59]. Carnauba wax coatings can reduce transpiration water loss in citrus fruits, maintain peel firmness, and delay tissue softening [60]. These findings indicate that the further introduction of essential oils or fruit and vegetable by-product extracts can enhance antimicrobial and antioxidant preservation effects [61]. In addition, wax-based materials can be used for hydrophobic modification of paper-based packaging and hydrophilic biofilm surfaces [62,63].

### 4.2. Oleogel-Based Gels

Oleogels are semi-solid lipid gel systems formed by immobilizing a large amount of liquid oil with a small amount of gelator. They usually use vegetable oils, waste cooking oils, or functional oils as the continuous phase, and natural waxes, ethyl cellulose, monoglycerides, phytosterols, lecithin, or biopolymer aerogels as gelators. Three-dimensional networks can be formed through crystallization, self-assembly, polymer chain entanglement, or confinement by porous frameworks [64,65]. In food packaging, oleogels can improve the hydrophobic barrier properties of materials and serve as reservoirs for essential oils, carotenoids, polyphenols, and other hydrophobic natural extracts, enabling stable loading and slow release of active components. Their gelation mechanisms vary with the type of gelator. Wax-based oleogels rely on cooling-induced wax crystallization to construct crystal networks; ethyl cellulose oleogels mainly form polymer networks through hydrophobic interactions, chain entanglement, and hydrogen bonding; and protein, polysaccharide aerogel, or emulsion-templated oleogels depend on capillary absorption and spatial confinement of the oil phase within porous frameworks. Studies have shown that oregano-enriched oleogels introduced as the oil phase into sodium caseinate edible films can reduce film solubility and water vapor permeability and promote the gradual release of antioxidants, thereby helping control moisture evaporation and lipid oxidation in dried meat packaging [65]. Ethyl cellulose oleogels can also form gel-state biodegradable thermoplastic materials with certain mechanical strength, environmental stability, and processability. For example, such materials can be processed at 130–165 °C, and the oil/polymer ratio can be adjusted from 10:3.5 to 1:2, indicating their potential as lipid-based packaging materials [66]. However, oleogel packaging still faces challenges such as oil-phase migration, insufficient gel stability, relatively high processing temperatures, and limited compatibility with hydrophilic biopolymers. Future research should focus on food-grade low-temperature gelation, emulsion-template stabilization, and multilayer structural design to improve controllability in active and intelligent packaging applications [67].

However, lipid-based gel packaging is not a universal solution. Wax coatings and oleogels usually have limited mechanical strength, weak adhesion to some hydrophilic substrates, possible oil migration or crystallization instability, and sensitivity to temperature fluctuations. High levels of waxes or essential oils may also affect transparency, sensory quality, heat-sealing behavior, and regulatory acceptance. Therefore, lipid-based gels are more suitable as barrier or active-release layers in multilayer systems than as independent structural packaging materials.

## 5. Other Natural Extract-Based Gels

In addition to typical polysaccharide-, protein-, and lipid-based gel matrices, other naturally derived materials, such as lignin, tannic acid-based metal-phenolic networks, shellac resin, cutin, and suberin, can also be used in food packaging systems. These materials are obtained from lignocellulosic or food-processing residues and lac insect secretions, and contain aromatic structures, phenolic hydroxyl groups, ester bonds, long-chain aliphatic structures, or natural resin structures. Although they do not necessarily form conventional hydrogels, these materials can form cohesive coatings or functional interfaces through non-covalent association, metal–phenolic coordination, or covalent crosslinking. Lignin is an abundant aromatic natural polymer with antioxidant, antimicrobial, and UV-absorbing properties [68,69,70]. Nanosizing lignin can improve its dispersibility and functional efficiency in biobased packaging matrices, and it can also be used to load anthocyanins, improve antioxidant performance, and extend the shelf life of fruits and vegetables. For example, lignin nanoparticles can encapsulate anthocyanins with an efficiency of up to 92.32%, and polyvinyl alcohol/polyethylene glycol films containing lignin-nanoencapsulated anthocyanins can slow tomato quality deterioration during 15 d of storage at room temperature [70]. Tannic acid and other plant polyphenols can form metal-phenolic networks through hydrogen bonding, π-π stacking, and coordination with metal ions such as Fe^3+^, Zn^2+^, and Ca^2+^, providing interfacial adhesion, antioxidant, antimicrobial, and pH-responsive properties [71,72,73]. Shellac is a natural resin with good film-forming ability, gloss, and moisture resistance, and it can be combined with zein, curcumin, limonin, or resveratrol to construct hydrophobic active coatings and preservation films [74,75,76]. Cutin and suberin are plant-derived natural polyesters whose native function is to protect plant tissues from water loss and external contamination; therefore, they have potential as barrier coatings for fiber-based packaging. Birch-bark suberin aqueous dispersion-coated paperboard can achieve a water vapor transmission rate of 18 g/m^2^/day [77,78,79].

Recent primary studies further clarify how these less conventional materials function in packaging. Armored lignin nanoparticles embedded in electrospun cellulose acetate membranes improved light shielding, antioxidant, antimicrobial, and oxygen and water-vapor barrier performance and extended apple shelf life by at least 3 d [80]. For metal-phenolic networks, procyanidin-Zn^2+^ particles incorporated into tilapia-gelatin films increased tensile strength to 82.47 MPa and extended pork shelf life by 5 d relative to the control [81]. These systems therefore act mainly as reinforcing and active domains within a continuous biopolymer matrix rather than as stand-alone gels.

A distinct emerging matrix is the supramolecular hydrogel formed directly by natural small molecules. Glycyrrhizic acid can self-assemble into hydrogen-bonded nanofibrils; coupling this network with Ca^2+^-crosslinked sodium alginate produced a robust, pH-responsive double-network hydrogel for controlled nutrient release [82]. Glycyrrhizic acid and berberine were also co-assembled through hydrogen bonding and electrostatic interactions into a sprayable, transparent, UV-blocking, and washable hydrogel. Under white light, the system generated reactive oxygen species and inhibited *E. coli* and *S. aureus* while maintaining fruit quality during storage [83]. In contrast to extract-loaded films, the natural active compounds in these systems participate directly in network construction.

Recent studies also provide quantitative evidence for natural resin and plant-polyester barrier systems. Rosin resin reduced water solubility and improved UV, water-vapor, and oxygen barrier properties in chitosan composite films [84]. On bagasse paperboard, two layers of 40% shellac reduced water-vapor and oxygen permeability by 82.2% and 99.5%, respectively, compared with uncoated paperboard [85]. A wood-derived suberin/cellulose nanofiber dispersion formed self-standing films and fruit coatings with UV shielding, antimicrobial activity, and an increase in water contact angle from 61° to 83° [86]. These results support the use of resins and plant polyesters as hydrophobic coating phases, although a supporting polymer or fibrous network remains important for film integrity and dispersion stability.

Despite these advantages, other naturally derived gel-like systems remain less mature than polysaccharide, protein, and lipid matrices. Their composition is often heterogeneous, and extraction, purification, and functional standardization may increase cost. These systems are therefore promising mainly as functional interfaces, hydrophobic coatings, or specialty barrier layers rather than as fully developed stand-alone packaging matrices.

To complement the representative examples discussed above, Table 1 provides a cross-matrix comparison of the four major natural extract-based gel systems in terms of gelation mechanism, hydrophobic/barrier and mechanical performance, degradability, cost, advantages, limitations, and suitable food scenarios.

## 6. Structure–Function Mechanisms

Section 2, Section 3, Section 4 and Section 5 discuss natural extract-based gel packaging systems according to matrix type, whereas Table 1 compares their major material features. To avoid treating these systems as isolated examples, this section further integrates them from a structure-function perspective. Across polysaccharide, protein, lipid, and other naturally derived gel systems, packaging performance is mainly determined by three linked aspects: network construction, active-component retention or release, and matching with the target food environment.

**Figure 3:**
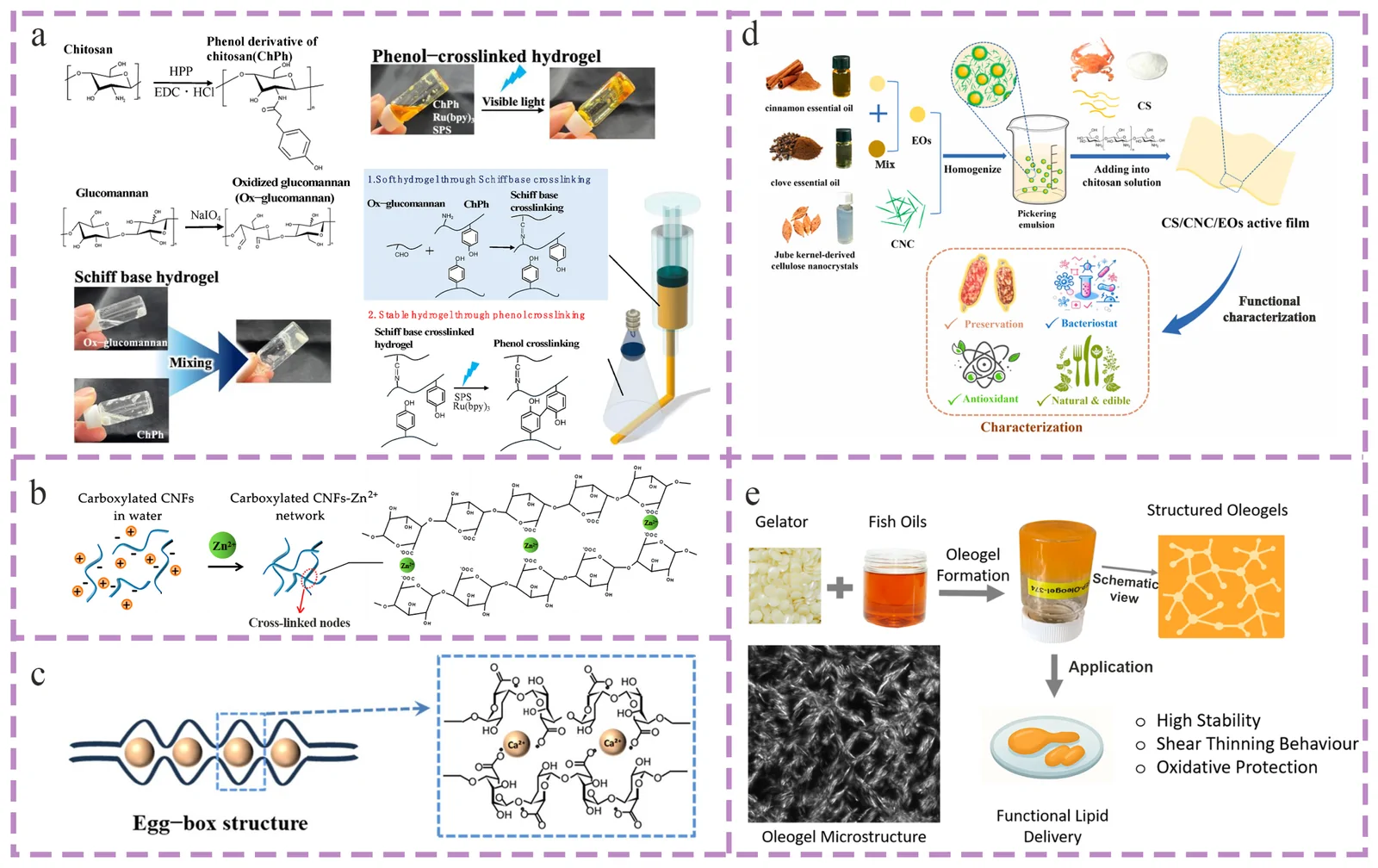
Representative gelation and functional construction mechanisms of natural extract-based multifunctional gels. (**a**) Schiff base and phenolic dual-crosslinking mechanism of chitosan/oxidized glucomannan composite hydrogel [87]. (**b**) Zn^2+^ crosslinked nanocellulose/anthocyanin hydrogel [88]. (**c**) Egg-box structure of metal ion-crosslinked alginate gels [89]. (**d**) Construction of cellulose nanocrystal-stabilized essential oil Pickering emulsion in chitosan films [90]. (**e**) Oleogel network formed by structuring fish oil with gelators [91].

At the network-construction level, matrix chains or gelators form three-dimensional structures through ionic crosslinking, hydrogen bonding, hydrophobic association, Schiff base reactions, lipid crystallization, or metal-phenolic coordination. Covalent and ionic bonds maintain network integrity, while hydrogen bonding, electrostatic attraction, and hydrophobic association promote chain aggregation and structural stabilization. Upon hydration, electrostatic or steric repulsion between polymer chains, together with osmotic pressure, drives water uptake and network expansion. The balance between these attractive and repulsive forces regulates the swelling degree, network pore size, free volume, swelling behavior, crystallinity, and interfacial compactness, thereby affecting mechanical strength, wet-state stability, adhesion, barrier properties, and molecular transport. Figure 3 summarizes representative mechanisms, including polysaccharide crosslinking, nanocellulose/anthocyanin hydrogels, Pickering-emulsion films, and oleogel networks.

At the active-component and application levels, natural extracts are stabilized and regulated by the gel matrix rather than simply mixed into it. Anthocyanins, curcumin, essential oils, tannic acid, flavonoids, and lignin-derived components interact with matrices through hydrogen bonding, electrostatic attraction, hydrophobic domains, π-π stacking, inclusion complexation, or metal coordination. Moderate interactions improve dispersion and reduce oxidation, volatilization, and leaching, whereas excessive binding may suppress release or weaken color-response sensitivity. Swelling-induced changes in mesh size can further regulate the diffusion and release of active compounds in response to pH, humidity, temperature, or volatile spoilage metabolites. Therefore, the gel network must also match food-specific spoilage pathways: high-moisture meat and aquatic products require humidity tolerance and volatile amine transport; fruits and vegetables require respiration and moisture regulation; and high-fat or dry foods require antioxidant release together with oxygen/light barriers.

Stimuli-responsive gels change their structure or color when exposed to pH, humidity, temperature, ions, or spoilage-related compounds. These changes may alter network swelling, permeability, and active-compound release. In meat and aquatic-product packaging, volatile amines can also induce visible color changes in anthocyanin- or curcumin-containing gels, allowing food freshness to be monitored.

**Figure 4:**
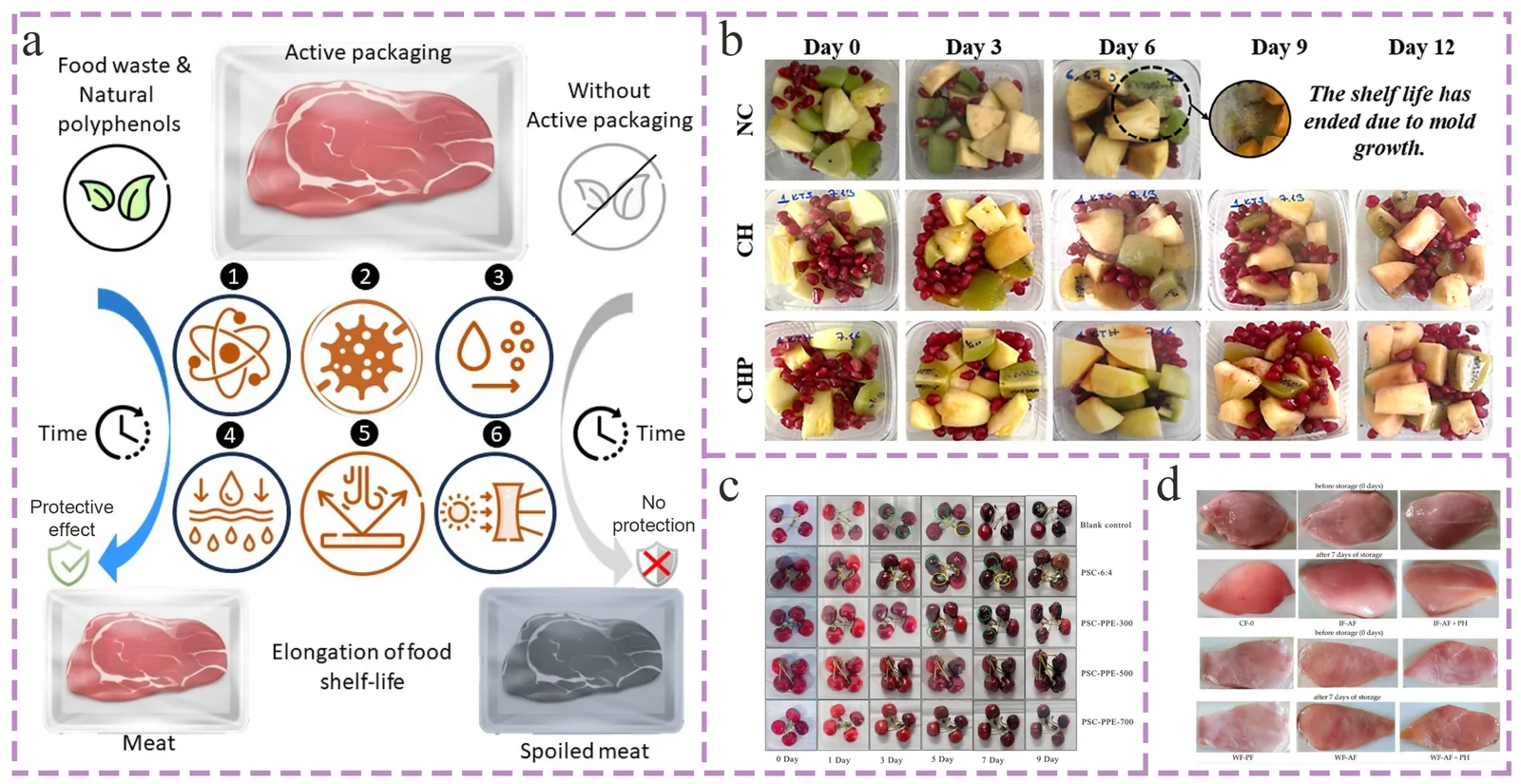
Representative preservation applications of natural active substance-loaded gel and coating packaging systems. (**a**) Extension of the shelf lives of food products using natural and food-waste polyphenols: (1) antioxidant property; (2) added antimicrobial property; (3) slow release of polyphenols; (4) improved water vapor permeability; (5) improved gas barrier property; (6) improved light barrier property [6]. (**b**) Fresh-cut fruit preservation by phenolic-enriched chitosan coating [92]. (**c**) Cherry preservation by anthocyanin-containing composite films [93]. (**d**) Chicken breast preservation by protein hydrolysate-containing alginate films [94].

Figure 4 illustrates representative preservation applications of natural active substance-loaded gel/coating packaging systems. Polyphenolic compounds can delay food quality deterioration through antioxidant and antimicrobial effects and improved barrier performance; among them, phenolic extract-reinforced chitosan coatings can better maintain the appearance quality of fresh-cut fruits. Natural pigments not only possess certain antioxidant activity but also endow packaging materials with color-responsive functions. Active components such as protein hydrolysates can improve the storage stability of refrigerated meat by inhibiting lipid oxidation and microbial growth. Overall, natural active substance-based gel packaging is evolving from single preservation functions toward multifunctional systems that integrate antioxidant activity, antimicrobial effects, barrier regulation, and quality monitoring.

## 7. Industrialization and Challenges

At present, industrialization-related studies on natural extract-based multifunctional gels mainly focus on edible coatings, cast films, indicator labels, aerogel pads, paper-based coatings, and multilayer composite films. Edible dipping or spraying coatings are closest to practical use for fruits and vegetables because they require simple equipment and can be integrated into existing postharvest handling lines, as shown by starch/beeswax and chitosan-phenolic coating systems [59,92]. Cast films and laminated coatings are more suitable for meat and aquatic products, where antimicrobial release, oxygen barrier properties, and volatile amine response are required [93,94]. Aerogel labels and electrospun films provide higher response sensitivity, but their scale-up is constrained by low production efficiency and equipment cost [14]. From a cost perspective, polysaccharide matrices such as starch, alginate, pectin, and chitosan are generally more affordable and available, whereas purified proteins, natural pigments, essential oils, nanofillers, and multilayer or freeze-dried structures can substantially increase formulation and processing costs [95]. Therefore, industrial development should evaluate not only functional performance but also raw-material availability, extraction cost, drying energy consumption, coating-line compatibility, and batch-to-batch reproducibility. Patent activity also reflects ongoing efforts toward practical application. A starch-seaweed-paper-fiber barrier coating (US9878839B2) and an edible chitosan/green-tea-polyphenol active coating disclosed in a Chinese patent application (CN103087356A) provide representative formulations for improving barrier performance and controlling antioxidant release.

However, the industrialization of these materials still faces multiple challenges. First, the insufficient stability of natural extracts remains a key limiting factor. Anthocyanins, curcumin, tea polyphenols, flavonoids, and essential oils are susceptible to light, oxygen, temperature, pH, and humidity, leading to discoloration, oxidation, volatilization, or activity loss. Strategies such as emulsion stabilization, microencapsulation, metal-phenolic coordination, and multilayer protection can improve stability, but they may also increase formulation complexity and preparation costs [95]. Second, the compatibility between natural extracts and gel matrices is not always ideal. Hydrophobic components such as essential oils, curcumin, and resins tend to aggregate in hydrophilic polysaccharide or protein matrices, leading to uneven dispersion, unstable release, and reduced mechanical properties. Therefore, interfacial compatibility design remains a core issue in practical applications. Another important challenge is the balance between functional performance and basic packaging properties. Many natural gel matrices are highly hydrophilic and tend to absorb water and swell under high-humidity conditions, resulting in decreased water vapor barrier performance and mechanical strength. Crosslinking, nano-reinforcement, hydrophobic modification, wax coating, and multilayer structures can improve water resistance; however, excessive crosslinking or overly strong hydrophobic modification may reduce transparency, color response speed, active substance release efficiency, and biodegradability. Therefore, natural extract-based gel packaging should not focus on a single performance indicator, but should comprehensively optimize mechanical strength, barrier properties, release behavior, response sensitivity, biodegradability, and food-contact safety. Natural gels generally offer higher renewable content and potentially faster degradation than persistent synthetic gels, whereas synthetic networks often provide greater hardness and dimensional stability; actual rates and stability depend on crosslink density, crystallinity, moisture, thickness, and disposal conditions.

For intelligent packaging, response accuracy and standardized evaluation remain prominent challenges during industrialization. Color changes in natural pigments are influenced not only by food spoilage, but also by humidity, oxygen concentration, the intrinsic color of food, light exposure, temperature fluctuations, and initial pH, which may lead to false-positive or false-negative judgments [96]. At present, many studies still use ammonia or buffer solutions to simulate spoilage environments, whereas the composition of volatile compounds in real food systems is more complex. Therefore, future studies should establish reliable correlations among color parameters, total volatile basic nitrogen, pH, total viable counts, lipid oxidation, sensory quality, and storage time to improve the practical judgment accuracy of intelligent gel packaging. Food-contact safety and regulatory assessment should also not be overlooked. Small-molecule active substances, metal ions, nanofillers, or crosslinking agents in packaging materials may migrate into foods, and migration can be affected by food composition, contact time, temperature, pH, and fat content [97]. Before food-contact commercialization, these materials should undergo standardized overall and specific migration tests, sensory evaluation, cytotoxicity and long-term toxicity assessments, degradation-product characterization, and real-food validation under relevant storage conditions. Meanwhile, the source, batch composition, and activity intensity of natural extracts may vary with plant variety, season, and extraction process; thus, stable raw material supply and quality control systems are still needed. Extracting natural pigments, polyphenols, pectin, starch, lignin, and waxes from agricultural by-products and food processing by-products is a feasible strategy for reducing cost and improving sustainability [98].

## 8. Conclusions and Outlook

Natural extract-based multifunctional gels should be regarded not simply as packaging materials containing natural extracts, but as structured gel systems that organize, stabilize, and regulate natural active components during food storage. By constructing renewable polysaccharide, protein, lipid, or other naturally derived networks, these gels can simultaneously provide mechanical support, barrier regulation, active-agent immobilization, controlled release, and freshness-responsive behavior. This review summarizes recent advances according to matrix type and emphasizes that each gel matrix contributes a distinct structural role: polysaccharide gels provide abundant functional groups and versatile pH-responsive platforms; protein gels offer diverse binding sites and oxygen-barrier potential; lipid gels contribute hydrophobic barriers and reservoirs for lipophilic active substances; and lignin, metal-phenolic networks, shellac, cutin, and suberin broaden the possibilities for surface functionalization and biomimetic barrier design. The performance of these systems is determined by the coupling of gel network architecture, extract chemistry, and food-specific mass transfer, rather than by the activity of natural extracts alone.

Although substantial progress has been made in recent years, the industrial application of natural extract-based gels is still limited by insufficient extract stability, poor water resistance of hydrophilic gel matrices, limited mechanical properties of some biodegradable films, inadequate accuracy of intelligent indication, and insufficient safety evaluation. Future research should shift from the preparation of single-function films to systematic packaging design for real food scenarios. For example, meat and aquatic products require strong antimicrobial capacity and reliable amine-responsive indication; fruits and vegetables require respiration regulation, moisture retention, and anti-browning functions; high-fat foods rely more on antioxidant activity and oxygen/light barrier properties; and high-moisture foods require better wet-state stability and structural integrity. Future development may proceed in four directions. First, the stability of natural extracts should be improved through microencapsulation, emulsion design, metal-phenolic coordination, and multilayer structural protection. Second, the water resistance, mechanical properties, and barrier performance of gel matrices should be enhanced through dynamic covalent crosslinking, ionic crosslinking, nano-reinforcement, hydrophobic interfacial modification, and interpenetrating network construction. Third, intelligent packaging should be combined with digital color analysis, smartphone recognition, and predictive models to improve the objectivity and accuracy of freshness monitoring. Fourth, food-grade raw materials, agricultural by-product resources, and scalable green processing technologies should be used to establish an integrated system covering raw materials, preparation, application, and degradation evaluation. Overall, natural extract-based multifunctional gels combine sustainability and functionality and hold important application prospects in future high-performance, biodegradable, and intelligent food packaging.

## Figures and Tables

**Table 1:** Comparative characteristics of major natural extract-based gel matrices for food packaging.

Matrix	Composition	Mechanism	Measured Property or Effect	Advantage	Disadvantage	Application	Study Reference
Polysaccharide	Chitosan/PVA; curcumin inclusion complexes	Hydrogen bonding and polymer-chain association	ABTS 98%; DPPH 87%; enhance antibacterial ability	Combined antioxidant, antimicrobial, and indicator functions	Hydrophilicity and active migration require control	Active and freshness-responsive film	Huang et al. (2024) [9]
	Alginate/anthocyanin/cellulose nanocrystal; beeswax coating	Casting plus hydrophobic surface coating; ionic and hydrogen-bonded matrix	Water contact angle 111.5°; 108.3° after 10 d at 100% RH	Enhance waterproofing performance	Added coating step may slow color response	Humidity-resistant indicator film	Shi et al. (2023) [19]
	Pectin/whey protein; purple sweet potato extract	Composite casting; protein-polysaccharide association	Lowest solubility and water-vapor permeability at 500 mg/100 mL extract	Dual preservation and pH indication	Moisture and allergen considerations	Grape preservation and shrimp freshness monitoring	Ke et al. (2025) [29]
	Starch/PVA; *Clitoria ternatea* extract	Casting; gelatinization and hydrogen-bonded blending	About 70% average mass loss during composting	Renewable matrix with extract release and compostability	Starch brittleness and water sensitivity	Antioxidant pH-sensitive packaging	do Nascimento et al. (2024) [34]
Protein	Soy protein isolate; *Zanthoxylum bungeanum* leaf extract	Solution casting; heat-induced protein association and polyphenol-protein interactions	Tensile strength 12.95 MPa; elongation at break 85.33% at 5% extract	Enhance mechanical properties and antioxidant capacity	Moisture sensitivity and allergen labeling require consideration	Active film for cherry tomato preservation	Yu et al. (2023) [53]
	Chitosan/soy protein isolate; pistachio-hull carbon dots and anthocyanins	Solution casting; polymer association with nanofiller-pigment interactions	ABTS 82.3%; DPPH 90.6%; fish shelf life extended to 12 d at 4 °C	Integrates antioxidant, antimicrobial, and colorimetric functions	Moisture sensitivity and carbon-dot migration require evaluation	Fish freshness monitoring and preservation	Hadavifar et al. (2025) [56]
Lipid	Carnauba wax-based edible coating	Emulsification, surface application, drying, and wax crystallization	Reduced transpiration and weight loss; maintained peel firmness and delayed softening	Direct hydrophobic barrier for fresh fruit	Coating uniformity, gas exchange, and sensory effects require control	Postharvest Moro orange storage	Babarabie et al. (2024) [60]
	Sodium caseinate film; oregano-enriched oleogel as the oil phase	Film casting with oleogel incorporation; lipid self-assembly within a protein matrix	Reduced film solubility and water-vapor permeability; gradual antioxidant release	Moisture barrier combined with controlled antioxidant delivery	Oil migration and compatibility with the hydrophilic matrix require evaluation	Active packaging for dried meat	Sánchez-Ramírez et al. (2025) [65]
Other	PVA/PEG; lignin-nanoencapsulated agro-waste anthocyanins	Nanoparticle dispersion and hydrogen-bonded matrix	Encapsulation efficiency up to 92.32%; slowed tomato deterioration for 15 d	High active retention and by-product valorization	Extraction standardization and migration data remain limited	Active tomato packaging	Meenu et al. (2025) [70]
	Glycyrrhizic acid/berberine	Small-molecule co-assembly; hydrogen bonding and electrostatic interactions	Transparent, UV-blocking, washable, and light-activated antibacterial hydrogel	Natural active molecules form the gel network directly	Performance depends on light exposure; removal and migration require validation	Sprayable coating for perishable fruits	Sun et al. (2025) [83]
	Wood-derived suberin/cellulose nanofibers	Nanofibrillar reinforcement and stabilization	Water contact angle increased from 61° to 83°; UV shielding and antimicrobial activity	By-product-derived hydrophobic and active barrier	Requires a supporting fibrous network and food-contact migration data	Banana coating and strawberry packaging	Qasim et al. (2024) [86]

The comparison is based on the representative studies summarized in Section 2, Section 3, Section 4 and Section 5. Practical suitability may vary with formulation, processing method, food composition, and storage conditions.

## Data Availability

No new data were created or analyzed in this study. Data sharing is not applicable to this article.
